# Hidden IgG Antibodies to the Tumor-Associated Thomsen-Friedenreich Antigen in Gastric Cancer Patients: Lectin Reactivity, Avidity, and Clinical Relevance

**DOI:** 10.1155/2017/6097647

**Published:** 2017-02-21

**Authors:** Oleg Kurtenkov, Kersti Klaamas

**Affiliations:** Department of Oncology and Immunology, National Institute for Health Development, Hiiu 42, 11619 Tallinn, Estonia

## Abstract

Natural antibodies to the tumor-associated Thomsen-Friedenreich antigen (TF) are related to tumor immunosurveillance and cancer patients' survival. Hidden IgG antibodies (HAbs) to TF, their lectin reactivity, avidity, and clinical relevance were studied. HAbs were present in cancer patients and controls. A decreased level of IgG HAbs was detected in cancer. The HAbs level positively correlated with the sialospecific SNA lectin binding in purified total IgG (tIgG) in donors and cancer patients, indicating that HAbs are higher sialylated. The avidity of anti-TF IgG in tIgG samples was lower in cancer patients (*P* = 0.025) while no difference in the avidity of free anti-TF IgG was established. A negative correlation between the avidity of anti-TF IgG in tIgG and SNA binding in both groups was observed (*P* < 0.0001). The HAbs level negatively correlated with the anti-TF IgG avidity in tIgG only in donors (*P* = 0.003). Changes in the level of HAbs and Abs avidity showed a rather good stage- and gender-dependent diagnostic accuracy. Cancer patients with a lower anti-TF IgG avidity in tIgG showed a benefit in survival. Thus the TF-specific HAbs represent a particular subset of anti-TF IgG that differ from free serum anti-TF IgG in SNA reactivity, avidity, diagnostic potential, and relation to survival.

## 1. Introduction

The expression of posttranscriptionally modified carboepitopes is a common feature of malignant cells. The Thomsen-Friedenreich disaccharide Gal*β*1-3GalNAc*α*/*β*-O-Ser/Thr (TF, CD 176) is overexpressed in a majority of adenocarcinomas [1–4] including gastric cancer which is considered one of the most deadly tumors worldwide. TF is also expressed on cancer stem cells [5] and is a marker of cancer-initiating cells [6]. Since the 1980s it has been demonstrated that TF- and *α*Gal-glycotope- (Gal*α*1,3-Gal*β*-) specific Abs appear early after birth and seem to be induced by intestinal microflora [7, 8].

The presence of naturally occurring TF-specific autoantibodies of different subclasses in cancer is a well-documented fact [9–12]. Moreover, the level of anti-TF antibodies (Abs) in the circulation is usually decreased in cancer, which is associated with tumor progression and patient survival [4, 11, 13] suggesting the important role of anti-TF Abs in antitumor immunity. The TF and endothelium-expressed galectin-3 have been identified as important molecular mechanisms initiating tumor/endothelial cell adhesion and metastasis [14, 15]. It has been shown that treatment of mice with a monoclonal anti-TF IgG Abs JAA-F11 inhibited lung metastasis and improved prognosis in a mouse breast cancer model [16] indicating that anti-TF humoral immune response has a therapeutic potential.

There is evidence that an appreciable amount of Abs is present in the circulation in a bound form, which phenomenon is called “hidden antibodies” (HAbs), and remains undetectable by conventional serologic methods [17, 18]. No special studies have been performed so far on the role of hidden IgG Abs to tumor-associated antigens in cancer. Recently we have demonstrated that anti-TF Abs in the serum (a pool of all Ig isotypes) of patients with gastric cancer show a significantly higher reactivity to sialic acid-specific* Sambucus nigra* lectin (SNA) than in controls [19]. Moreover, the SNA-reactive anti-TF IgG Abs demonstrated a higher avidity in cancer [20]. These findings suggest that an altered glycosylation of anti-TF natural Abs may be used as a serologic biomarker for cancer.

In the present study we show that, in contrast to xenogeneic naturally occurring anti-*α*Gal IgG, an appreciable amount of natural anti-TF IgG Abs is present in the circulation in a hidden form in both cancer patients and controls. HAbs showed a decreased level in cancer and exhibited a distinct SNA lectin reactivity, avidity, and relation to survival of gastric cancer patients indicating that TF-specific HAbs represent a particular subset of anti-TF IgG Abs which deserves further study to specify their clinical importance.

## 2. Material and Methods

### 2.1. Subjects and Samples

Serum samples were obtained from healthy blood donors (*n* = 28) and patients with histologically verified gastric carcinoma (*n* = 41) (Table 1). The investigation was carried out in accordance with the ICH GCP Standards and approved by the Tallinn Medical Research Ethics Committee, Estonia. A written informed consent was obtained from each subject. Tumor staging was based on the histopathological (pTNM) classification of malignant tumors. Serum samples were stored in aliquots at −20°C until use.

### 2.2. The Purification of Serum Total IgG

The purification of serum total IgG (tIgG) was performed on the Protein G HP Spin Trap column as described by the manufacturer (GE Healthcare, USA). The tIgG samples were eluted at pH 2.5, immediately neutralized, dialyzed against phosphate buffered solution (PBS, 0.1% NaN_3_), and stored at +4°C until being tested. About 8.5 mg of IgG was obtained from 1 mL of serum applied onto the Protein G Sepharose column. To obtain the IgG-depleted serum we used the same method on the Protein G HP Spin Trap column, except the serum volume applied to the Protein G column was three times lower, and the complete depletion of IgG was controlled using the Easy-Titer IgG Assay Kit (Thermo Scientific, USA).

### 2.3. The TF-Specific Antibody Assay

The anti-TF and anti-*α*Gal IgG antibody levels were determined by enzyme-linked immunosorbent assay (ELISA) as described elsewhere [19, 21] with minor modifications. Briefly, the plates (Maxisorp, Nunc, Denmark) were coated with a synthetic TF*α*- or *α*Gal-polyacrylamide conjugate (Lectinity, Russia) in carbonate buffer, pH 9.6, 5 *μ*g per well. After overnight incubation at +4°C, triple washing, and blocking with Superblock solution (Pierce, USA) for 15 min at 25°C, the serum or purified IgG samples diluted to 1 : 25 in PBS-0.05% Tween (Tw) were applied for 1.5 hr at 25°C. The concentration of IgG in serum and tIgG samples was measured by the Easy-Titer IgG Assay Kit (Thermo Scientific, USA) and the IgG concentration in the tIgG probe adjusted to that in serum. After subsequent washing with PBS-Tw, the bound anti-TF or anti-*α*Gal IgG was detected with alkaline phosphatase conjugated goat anti-human IgG (Dako, Denmark) and p-nitrophenylphosphate disodium hexahydrate (Sigma, USA). The absorbance values were read at 405 nm (Tecan Reader, Austria).

### 2.4. The Hidden Anti-TF IgG Antibody Level and the Impact of IgG-Depleted Serum

The IgG concentration in tIgG samples was adjusted to that in serum as described above and the level of anti-TF IgG Ab in serum and tIgG samples was measured by ELISA. The level of hidden IgG antibodies (HAbs) was calculated as the difference between the levels of anti-TF IgG (OD values) in tIgG and serum.

To evaluate the impact of IgG-depleted serum the purified tIgG samples of cancer patients (*n* = 5) and controls (*n* = 5) were incubated with an equal volume of the autologic IgG-depleted serum diluted from 1 : 10 to 1 : 100, or bovine serum albumin (BSA, 0.5–2,0 mg/mL) for 15 min at 25°C, and the HAb levels (mean OD value) after incubation with PBS-BSA or after addition of IgG-depleted serum dilutions were determined as described above and presented in Figure 3.

### 2.5. The Reactivity of Anti-TF and Anti-*α*Gal Antibodies to* Sambucus nigra* agglutinin (SNA) and Concanavalin A (ConA) Lectin

The lectin reactivity of the TF-specific IgG was measured by ELISA in a similar way, except that the binding of neuraminic acid- (sialic acid-) specific* Sambucus nigra *agglutinin (SNA) and mannose-specific concanavalin A (ConA) to the absorbed serum anti-TF Abs (all isotypes) or anti-TF IgG from tIgG samples was measured as described elsewhere [19, 21]. Biotinylated SNA (Vector Laboratories Inc., USA) in 10 mmol/L HEPES, 0.15 mol/L NaCl, and 0.1 mmol/L CaCl_2_, pH 7.5, and biotinylated ConA (Sigma, USA) in the ConA binding buffer (0.05 mol/L Tris-HCl buffer, pH 7.2, containing 0.2 mol/L NaCl and 3 mmol/L CaCl_2_, MgCl_2_, and MnCl_2_) were both applied at a concentration of 5 *μ*g/mL each, for 1.5 h at 25°C. The bound lectins were detected with a streptavidin-alkaline phosphatase conjugate (Dako, Denmark) and p-nitrophenylphosphate (Sigma, USA). The optical density value of control wells (no sample) was subtracted from that of Ab-coated wells to determine the lectin binding. Each sample was analyzed in duplicate.

### 2.6. The Avidity of TF-Specific Antibodies

The assay is based on the dissociation of Ab-Ag complexes by 1.25 M ammonium thiocyanate (NH_4_SCN). This concentration of the chaotrope was selected in the preliminary titration experiments. Under the conditions used, about 60% of serum IgG antibodies of controls were detached after treatment with 1.25 M thiocyanate. From each sample, a series with and without thiocyanate treatment were made.

Purified total IgG samples with an adjusted IgG concentration and serum samples diluted to 1 : 25 were added to the wells coated with TF-PAA or *α*Gal–PAA glycoconjugate and blocked with the Superblock solution (Pierce, USA). After incubation for 1.5 hr at 25°C the wells were exposed to either PBS-0.05% Tw or NH_4_SCN solution (1.25 mol/L) for 15 min at +25°C. After triple washing, the wells were incubated with alkaline phosphatase-labeled anti-human IgG for 60 min at 37°C. The bound antibodies were detected with alkaline phosphatase conjugated goat anti-human IgG (Sigma, USA) and p-nitrophenylphosphate (Sigma, USA). The results were expressed as a relative avidity index (AI) representing the percentage of reactivity remaining in the thiocyanate treated well.

### 2.7. Statistical Analysis

Comparisons between the groups were performed using the nonparametric Mann–Whitney* U *test for unpaired data or Student's *t*-test, where appropriate, and the Pearson two-tailed correlation. A *P* ≤ 0.05 value was considered statistically significant. Survival analysis was carried out by the Kaplan-Meier method using the Estonian Cancer Registry database. The group median was used as a cut-off limit. The differences between cancer patients and controls in Ab levels and the avidity were evaluated for the diagnostic accuracy for cancer by the receiver operator characteristic (ROC) curve analysis. All calculations were performed using the GraphPad Prism 5 and SPSS 15.0 software.

## 3. Results

### 3.1. Anti-TF IgG, Anti-*α*Gal IgG, and Hidden Antibody Levels in Cancer Patients and Controls

**Figure 1:**
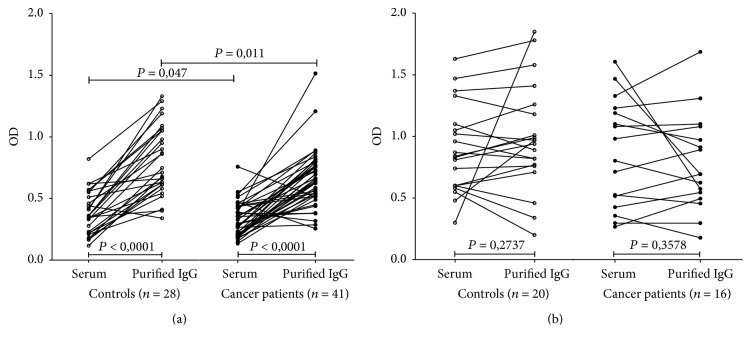
The level of anti-TF and anti-*α*Gal IgG antibodies in serum and total purified IgG. (a) The TF-specific antibody level (OD) in cancer patients and controls. Each dot represents one individual. The values in serum and purified tIgG for each individual are connected with lines. (b) Anti-*α*Gal IgG antibody levels. *P* values were calculated by the Mann–Whitney *U* test and are shown for significant differences.

Decreased anti-TF IgG levels were found in both the serum and purified tIgG samples (*P* = 0.047 and 0.011, resp.) in cancer patients compared to controls. The OD values were significantly higher in purified tIgG than in serum samples in donors (*P* < 0.0001) and cancer patients (*P* < 0.0001) (Figure 1(a)). Only one subject of 28 controls and three of 41 cancer patients (all stage 3 patients) showed a slightly lower level of anti-TF IgG in tIgG than in serum. No significant difference in anti-*α*Gal IgG level in the serum and purified IgG samples between patients and controls was observed (Figure 1(b)).

**Figure 2:**
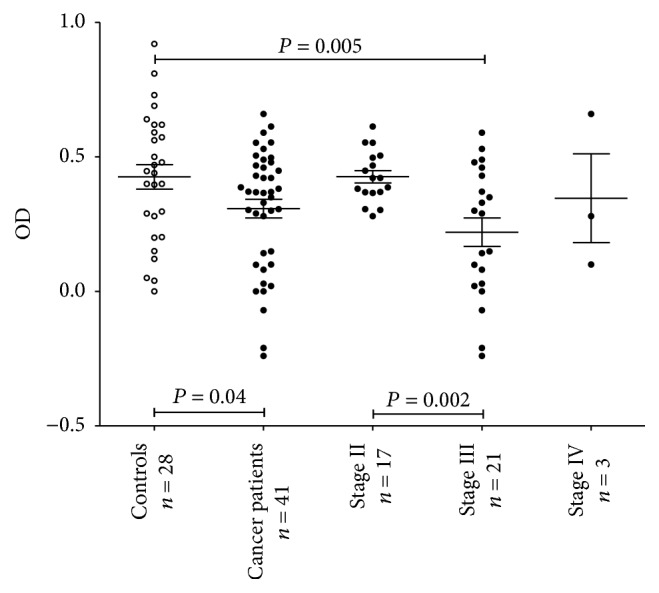
The level of TF-specific hidden IgG antibodies (HAbs) in controls and cancer patients by stage. Each dot represents one individual and group mean ± SEM is indicated by horizontal lines. The HAb level was calculated as the difference between the levels (OD values) of anti-TF-IgG in tIgG and serum. *P* values are shown for significant differences.

The level of hidden antibodies (HAb) defined as the difference between the levels in tIgG and serum was lower in cancer patients (*P* = 0.04). This decrease was mostly associated with stage 3 patients unlike controls (*P* = 0.005) (Figure 2) and related mainly to females (mean ± SEM: 0.19 ± 0.074; *P* = 0.01) compared with males (0.37 ± 0.058, *P* = 0.74). In contrast to anti-TF IgG, the level of hidden anti-*α*Gal IgG Abs was very low and showed no difference between controls and patients (mean OD ± SEM: 0.01 ± 0.09 and 0.09 ± 0.08 for donors and cancer patients, resp., *P* = 0.36). In eight of 20 controls and seven of 16 patients the level of anti-*α*Gal IgG in tIgG was even lower than in serum samples.

**Figure 3:**
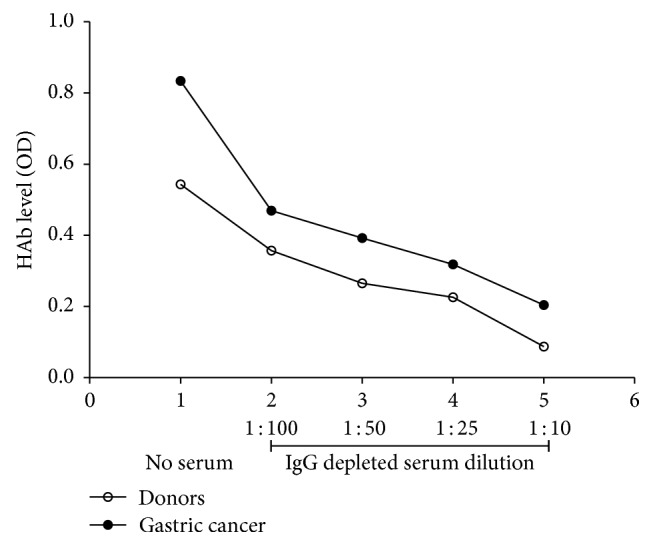
The effect of IgG-depleted serum on the level of anti-TF HAb in cancer patients and controls. The purified tIgG of cancer patients (*n* = 5) and controls (*n* = 5) was incubated with autologic IgG-depleted serum diluted from 1 : 10 to 1 : 100 for 15 min at 25°C. The level of anti-TF hidden antibodies was determined as described in Material and Methods; the mean value of HAb levels (OD) before and after addition of IgG-depleted serum dilutions is presented.

It is notable that after incubation of purified tIgG with IgG-depleted autologic serum the level of anti-TF IgG HAbs decreased dramatically and dose-dependently nearly down to the level in the serum already at the 1 : 10 dilution of the IgG-depleted serum (Figure 3). The impact of BSA addition (0.5–4 mg/mL) instead of IgG-depleted serum on the HAbs level was negligible being always below 10%. In addition, no such effect was observed with anti-*α*Gal Abs where the HAbs level was very low.

Thus, rather a high level of hidden anti-TF IgG Abs but not anti-*α*Gal hidden IgG was demonstrated in both gastric cancer patients and controls. A decreased level of anti-TF IgG HAb was observed in gastric cancer patients, especially in stage 3 females. It appears that the autologic IgG-depleted serum contains ligands that react with anti-TF IgG Abs, making them undetectable in the serum.

### 3.2. Anti-TF Antibody SNA and ConA Lectin Reactivity

**Figure 4:**
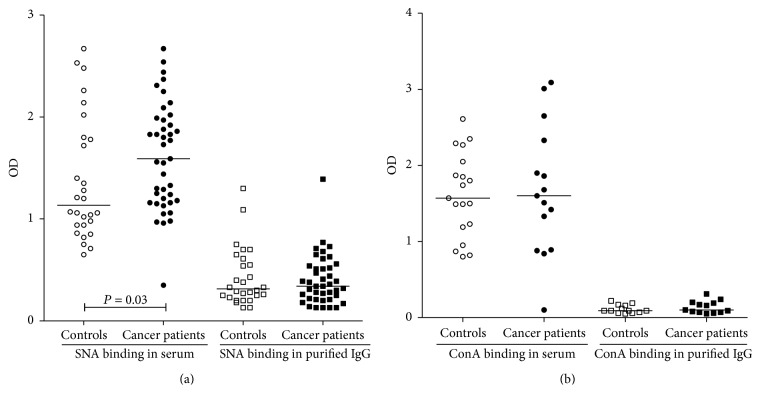
The binding of SNA (a) and ConA lectin (b) to anti-TF antibodies in serum and purified tIgG samples of cancer patients and controls. Each dot represents one individual and group median is indicated by horizontal lines. *P* values are shown for significant differences.

The levels of SNA and ConA binding to anti-TF Abs were much higher in serum than in total IgG samples (*P* < 0.0001) in patients and controls (Figures 4(a) and 4(b)), suggesting a higher sialylation of anti-TF IgM and/or IgA compared to IgG. A significantly higher SNA binding to serum anti-TF Abs in the cancer patients group was observed (mean OD ± SEM: 1.61 ± 0.08 and 1.37 ± 0.11; in patients and controls, resp., *P* = 0.03, Mann–Whitney test), whereas no difference between patients and controls was found for tIgG samples (mean OD ± SEM: 0.40 ± 0.053 and 0.426 ± 0.038, resp., *P* = 0.70).

**Figure 5:**
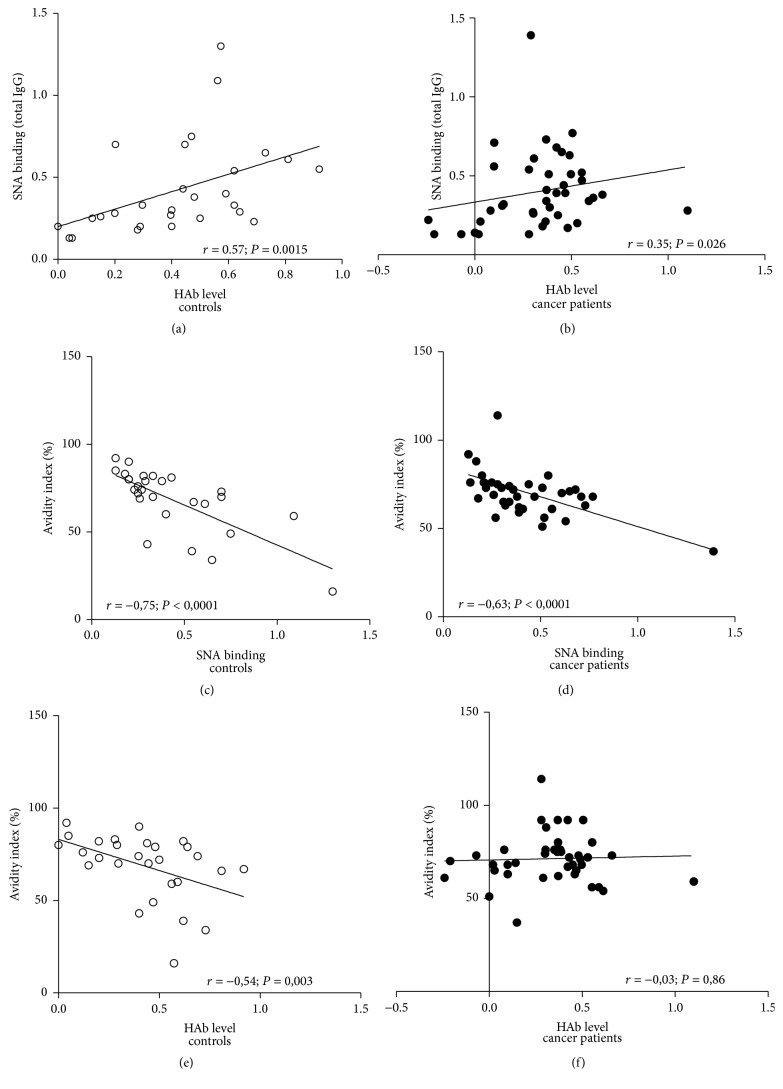
Correlation between the level of hidden TF-specific IgG antibodies, SNA binding, and the avidity of anti-TF IgG antibodies in gastric cancer patients and controls. (a, b) Correlation between the SNA binding to TF-specific IgG in purified tIgG and the HAb level; (c, d) correlation between the SNA binding and the avidity of TF-specific IgG in purified tIgG; (e, f) correlation between the HAb level and the avidity of TF-specific IgG in purified tIgG.

The ConA lectin binding to anti-TF Abs in serum and tIgG samples did not show a significant difference between patients and controls. The SNA binding to anti-TF IgG in purified tIgG samples positively correlated with the level of HAbs in donors (*r* = 0.572, *P* = 0.0015) and, to a lesser extent, in cancer patients (*r* = 0.347, *P* = 0.027) (Figures 5(a) and 5(b)), indicating that anti-TF hidden antibodies in purified tIgG are higher sialylated, especially in controls. No correlation between the SNA or ConA binding and anti-*α*Gal or anti-*α*Gal HAb levels was found.

### 3.3. The Avidity of Anti-TF IgG in Serum and Purified tIgG

**Figure 6:**
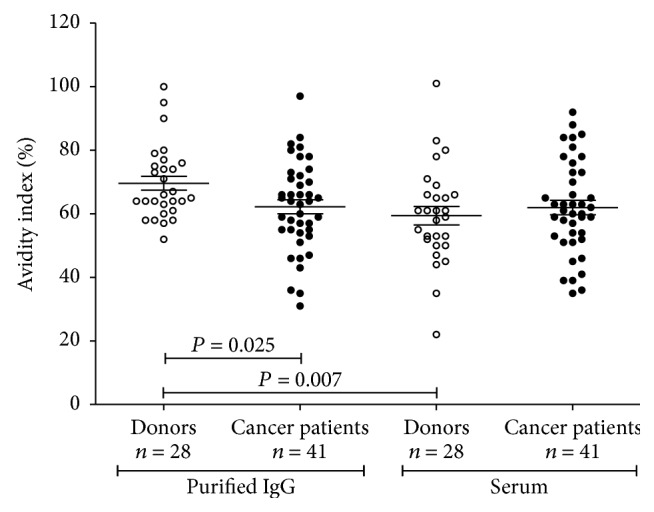
The avidity of TF-specific IgG antibodies in cancer patients and controls. The avidity indexes of anti-TF antibodies in serum and purified tIgG are compared. Each dot represents one individual and group mean ± SEM is indicated. *P* values were calculated by the unpaired Student's *t*-test and are shown for significant differences.

In donors, the avidity of anti-TF IgG Abs in purified tIgG was found to be higher than that in serum (*P* = 0.007), while no difference in cancer patients was observed (*P* = 0.80) (Figure 6). Moreover, the avidity of anti-TF IgG Abs in purified tIgG in donors was also higher than that in cancer patients (*P* = 0.025). The avidity of free serum anti-TF IgG was similar in patients and controls (*P* = 0.47).

The avidity of anti-TF IgG in tIgG negatively correlated with the SNA binding in both groups: *r* = −0.75 and *r* = −0.63 in controls and cancer patients, respectively (*P* < 0.0001) (Figures 5(c) and 5(d)). The avidity was lower in controls with a high level of HAbs (*r* = −0.54, *P* = 0.003), while no correlation between the HAbs level and IgG avidity was found in patients with cancer (*r* = −0.03, *P* = 0.86) (Figures 5(e) and 5(f)).

The data show that cancer patients differ from controls in HAbs avidity. Cancer patients reveal some deficiency of high-avidity TF-specific IgG in tIgG samples where HAbs are present.

### 3.4. Diagnostic Potential and Survival Analysis

**Figure 7:**
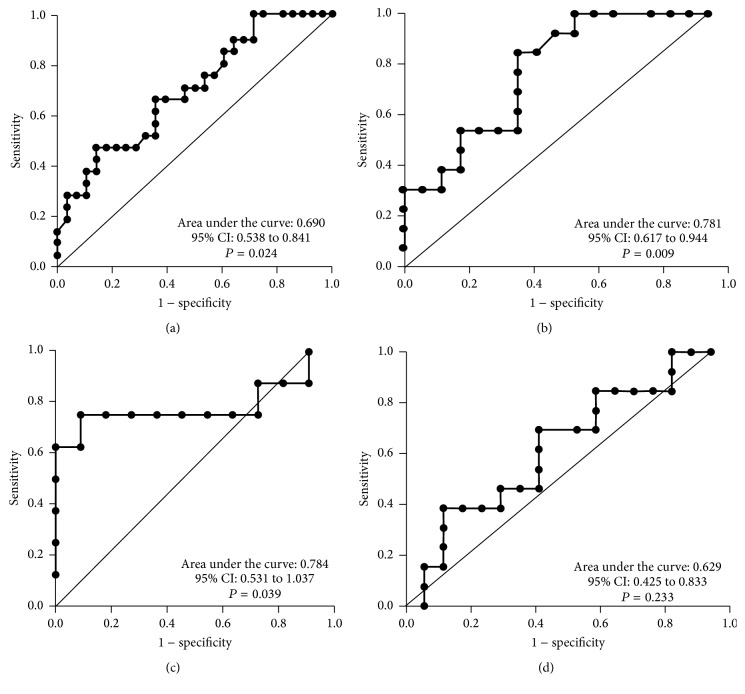
A receiver operator characteristic (ROC) curve analysis for the HAb level in stage 3 patients (a); in stage 3 females (b); and for the ratio anti-TF IgG avidity in tIgG/anti-TF IgG avidity in the serum of stage 3 males (c) and stage 3 females (d). The area under the ROC curve represents the diagnostic accuracy of the changes for gastric cancer.

Changes in the anti-TF IgG HAb level in the whole cancer group showed rather low accuracy (area under the ROC curve (AUC) = 0.61, 95% CI 0.47–0.75) as analyzed by ROC curve analysis. The sensitivity increased in stage 3 patients: AUC = 0.69 (95% CI 0.54–0.84) (Figure 7(a)). Interestingly, the HAb level showed higher diagnostic potential in females especially in stage 3 patients: area under the ROC curve 0.78 (95% CI 0.61–0.94, *P* = 0.0094) (Figure 7(b)), compared to males (AUC = 0.55, 95% CI 0.26–0.83).

The avidity of anti-TF IgG in purified tIgG samples also showed a moderate diagnostic value (AUC = 0.638, 95% CI 0.51–0.77, *P* = 0.052). A better discrimination of patients and controls was obtained by using the ratio: the avidity of anti-TF IgG in tIgG samples/the avidity of IgG in serum samples (AUC = 0.68, 95% CI 0.55–0.80, *P* = 0.010) especially in stage 3 males (AUC = 0.784, 95% CI 0.53–1.0, *P* = 0.04) (Figures 7(c) and 7(d)).

**Figure 8:**
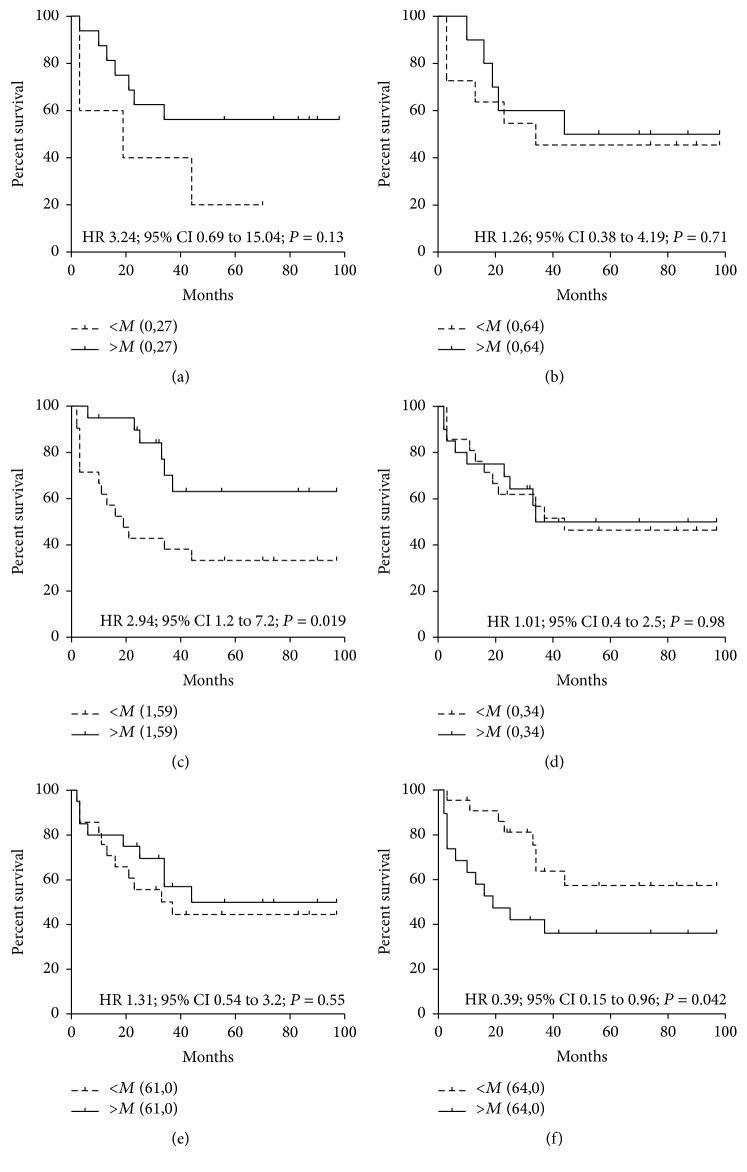
The probability of cancer patients survival in relation to anti-TF IgG level, SNA reactivity, and the avidity of TF-specific antibodies in serum and purified total IgG samples. Patients with either lower/equal (a dashed line) or higher values than median (a solid line) are compared using the Kaplan-Meier method. HR: hazard ratio with 95% confidence interval and *P* values are shown. (a) Anti-TF IgG level in serum (stage 3); (b) anti-TF IgG level in tIgG (stage 3); (c) the SNA binding to serum TF-specific antibodies; (d) the SNA binding to anti-TF IgG in tIgG; (e) the avidity of anti-TF IgG in serum; (f) the anti-TF IgG antibody avidity in tIgG and survival.

The higher level of anti-TF IgG in serum was associated with better survival in stage 3 patients (HR = 3.23 (0.69–15.0), *P* = 0.13), whereas no relation of this level in purified tIgG samples to survival was found (Figures 8(a) and 8(b)). Survival benefit was demonstrated by patients with a higher level of SNA binding to serum anti-TF Abs (a pool of all Ig isotypes): HR = 2.94 (1.2–7.2), *P* = 0.019. In contrast, the SNA reactivity of anti-TF IgG in tIgG samples showed no association with survival (HR = 1.01 (0.4–2.5), *P* = 0.98, Figures 8(c) and 8(d)). The level of HAbs had no association with survival. However, the higher avidity of anti-TF IgG in purified tIgG samples where HAbs were present (Figure 8(f)) was related to worse survival (HR = 0.439 (0.179–1.074), *P* = 0.07). This was mostly on account of stage 3 patients (HR = 0.34 (0.09–1.26), *P* = 0.10). No association of the avidity of serum anti-TF IgG Abs with survival (HR = 1.02 (0.43–2.41), *P* = 0.95) was established (Figure 8(e)).

In summary, the level of HAbs and the avidity of anti-TF IgG showed a moderate stage- and gender-dependent diagnostic accuracy. A better outcome was observed in patients with a high level of TF-specific IgG in serum (stage 3), in those with a high level of SNA binding to serum anti-TF Abs (a pool of all Ig isotypes), and in patients with a low avidity of anti-TF IgG antibodies in purified tIgG.

## 4. Discussion 

In this study, we focused on the anti-TF IgG HAb for several reasons. First, since the Fab fragment is not involved in the binding of IgG to PtG, the tIgG preparations are enriched in immune complexes, while the use of glycan- (TF-) specific sorbents for anti-TF Ab purification from the serum will exclude such a possibility due to the involvement of Fab fragments in the purification process. Second, the higher avidity of IgG allows the preferential binding of TF-positive ligands despite the possible interference of the other Ig isotypes. Third, using the Protein G it is much easier to purify IgG without the other Ig isotypes admixture. Fourth, the changes in the anti-TF IgG level showed a significant association with cancer progression and patients survival [11, 13] thus demonstrating a promising clinical potential. The possible role of the other isotypes of anti-TF Abs in the formation of HAbs needs a special study.

The results obtained in this work show that anti-TF IgG HAbs are present in the blood of both cancer patients and healthy controls. In contrast, no anti-*α*Gal HAbs or a very low amount of them was found in the same samples. Differently from controls a decreased level of anti-TF IgG HAbs was found in cancer patients (*P* = 0.005 for stage 3 patients).

It has been shown that the in vitro exposure of human IgG to protein-destabilizing chemical or physical factors results in the exposure of IgG “hidden” reactivity [17]. However, it remains unclear whether this exposure results from the dissociation of preexisting immune complexes of Abs with some ligands, the presence of IgG dimers, anti-idiotypic Abs, or the exposure of the polyreactivity/polyspecificity of hidden Abs [22–25]. The absence of *α*Gal-specific HAbs in the same purified tIgG samples strongly suggests that the purification of IgG per se is not a sufficient reason to explain the appearance of IgG HAbs. Moreover, the inhibition of the reactivity of hidden Abs by addition of IgG-depleted serum implies the role of serum-derived factors such as TF-positive or cross-reactive ligands that remain in the IgG-depleted serum and react with anti-TF IgG Abs again, making them HAbs. This restoration phenomenon (Figure 3) was absent in anti-*α*Gal IgG where a very low level of HAbs was detected, which indicates that no ligands for *α*Gal Abs are present in the IgG-depleted serum. We used anti-*α*Gal IgG as a distinctive control to show that the purification of IgG at acid pH is not the main reason behind changes in anti-glycan IgG reactivity. We suggest that the ligands for TF-specific Abs should be more informative and more specific for cancer than anti-TF Abs themselves or their subsets (glycoforms).

Immunoglobulins (Igs) are glycosylated molecules which display a set of glycoforms that differ in number, type, and site of oligosaccharide attachment [26]. The N-glycans of the Fc-fragment strongly influence IgG-Fc*γ* receptor interactions and thus the Fc-mediated effector mechanisms [27, 28]. Agalactosylated (asialylated) IgGs show an increased inflammatory activity that may promote tumor growth, while the Fc glycan sialylation may determine anti-inflammatory properties [29–31] though, besides IgG Fc sialylation, multiple mechanisms are involved in the anti-inflammatory effect of intravenous immunoglobulins [32]. It has also been shown that the anti-inflammatory potential of the sialylated IgG is mostly related to the SNA lectin-reactive sialic acids in the Fab fragment [33]. It is notable that the glycosylation of antigen-specific IgG can differ from total serum IgG glycosylation indicating that different B cell subsets produce differently glycosylated IgG [34, 35].

There is evidence that the aberrantly glycosylated serum IgG may be either of tumor origin, or accumulated in tumor tissue [36]. The higher levels of agalactosylated IgG oligosaccharides, which increase with tumor progression, were reported for patients with gastric, prostate, and ovarian cancers [36–38]. Up to now there are little data available on the glycosylation changes of Abs directed against antigens which are intrinsically involved in the pathogenesis of a specific disease, including cancer [21, 30, 36, 39–41].

The comparison of lectin binding patterns of anti-TF Abs in serum and tIgG shows that the main targets for binding SNA and ConA lectin in serum are TF-specific IgM and/or IgA. The SNA binding to free anti-TF Abs in serum was significantly higher in cancer patients (*P* = 0.03) (Figure 4(a)) whereas anti-TF IgG Abs in purified tIgG showed a similar SNA and ConA lectin binding in patients and controls. Since the SNA binding to TF-specific IgG positively correlated with the level of HAbs only in tIgG samples where both free and hidden anti-TF IgG were present, we conclude that HAbs are higher sialylated.

It is known that the total IgG purified on PtG sorbents is enriched by immune complexes and contains a lot of so-called “hitchhiker” ligands [42, 43]. We believe that this phenomenon is akin to the HAbs formation or is closely related to it, and many hitchhikers unidentified and untested as yet may be present in HAbs, including TF-positive ligands of tumor origin such as MUC1 which is overexpressed and aberrantly glycosylated in cancer, and identified as an antigenic component in IgG immune complexes in cancer patients [44, 45]. The modified self-components such as those derived from aged red blood cells may contain complexes of natural antibodies with TF-positive asialoglycophorin A or other senescent-associated epitopes connected to the clearance of aged erythrocytes [46, 47]. Thus the elimination of such complexes with natural Abs appears to be a normal physiological mechanism.

Contrary to blood donors, cancer patients demonstrated a lower avidity of anti-TF IgG in tIgG samples while no differences in the avidity of free serum TF-specific Abs between the two groups were observed. Since the tIgG contains both free Abs and HAbs, this decrease is obviously related to the HAbs that display a lower avidity in cancer.

The lower level of HAbs showed a rather good diagnostic accuracy for cancer in stage 3 females (AUC = 0.78) compared to males (AUC = 0.55). In contrast, the ratio of anti-TF IgG avidity in tIgG to the avidity of IgG in serum revealed a better diagnostic value in males (AUC = 0.784) than in females (AUC = 0.629) (Figure 7). Since the avidity of anti-TF IgG in serum showed no changes in cancer, the decrease of high-avidity anti-TF IgG HAbs in cancer patients, especially in males, may be of diagnostic value. At present we can give no explanation for these gender-related differences and further study is needed to evaluate the clinical utility of the above changes.

The SNA binding to anti-TF IgG in tIgG positively correlated with the HAbs levels in both groups (Figures 5(a) and 5(b)). At the same time, a negative correlation between the anti-TF Abs avidity in tIgG and the HAbs level was observed in controls (*r* = −0.54, *P* = 0.003) but not in cancer patients (*r* = −0.03, *P* = 0.86) (Figures 5(e) and 5(f)). These findings indicate that IgG HAbs are higher sialylated in cancer and display a lower avidity, which suggests that they represent a particular subset of TF- specific Abs.

It has been reported that the Fc glycan of adaptive IgG Abs is lower sialylated [48], though the vaccination in humans induced increased levels of antigen-specific IgG galactosylation and sialylation [35]. However, there are still no data about the sialylation of IgG-Fab fragments of tumor-related Abs in cancer. Notable is that the higher SNA reactivity of TF-specific Abs (a pool of all isotypes) in serum was associated with better survival (HR = 2.94 (1.2–7.2), *P* = 0.019), whereas no connection with the SNA reactivity of anti-TF IgG in tIgG (HR = 1.01 (0.4–2.5), *P* = 0.98) was demonstrated. We have shown recently that the higher avidity of SNA-reactive TF-specific Abs in the serum of cancer patients was not related to the IgG isotype [20]. In the present study, the higher avidity of anti-TF IgG in purified tIgG preparations containing HAbs was associated with worse survival (Figure 8(f)). In other words, a deficiency of high-avidity anti-TF IgG HAbs in cancer tIgG samples is associated with survival benefit, whereas the avidity of free serum TF-specific IgG was not related to survival.

It is to be noted that despite the TF expression on tumor cells there is no evidence that any adaptive humoral immune response to TF occurs in the tumor host. Instead, a decrease in the level of serum TF-specific Abs is usually observed [9–11, 13]. However, no data still exist on the possible involvement of any particular subset of anti-TF IgG Abs in this decrease. We found that healthy individuals demonstrated a significantly higher avidity of anti-TF IgG in tIgG samples, unlike gastric cancer patients whose avidity values in tIgG were low and similar to those in serum samples. We suggest that this may occur, at least in part, due to the elimination of high-avidity anti-TF Ab subset from the circulation of patients with cancer. This decrease of the avidity of naturally occurring anti-TF IgG Abs in the purified tIgG of patients with gastric cancer is, to our knowledge, described for the first time. We speculate that such a decrease may be a result of the interaction of these Abs with tumor-derived TF-positive ligands in situ or their increased clearance with circulating tumor cells. Which ligands are involved in the HAbs formation in cancer patients and healthy individuals remains to be elucidated.

In conclusion, TF-specific HAbs represent a particular subset of anti-TF IgG Abs that differ from free anti-TF IgG Abs in SNA reactivity, avidity, diagnostic potential, and relation to survival. Thus it appears that free serum anti-TF natural Abs in the circulation are only the “tip of the iceberg.” The hidden anti-TF antibody reactivity emerging in purified IgG suggests that serological testing with whole serum does not reflect the whole picture, and the HAbs analysis could tell us more about the other players and targets involved. Given that there are still no reliable biomarkers for gastric cancer, the changes in the level of anti-TF hidden IgG antibodies and their avidity found in this study merit further study. Whether the tumor-specific ligands are involved in the HAbs formation in patients with cancer remains to be clarified. The functional activity and clinically important effects of anti-TF Abs may apply to only a particular subset/glycoform of TF-specific Abs which contributes to cancer and its progression. Further characterization of TF-specific antibody subsets and potential ligands involved in the HAbs formation in health and disease may extend the concept of natural autoantibody signatures and improve the clinical utility of antibody-based diagnostic and predictive biomarkers.

## Figures and Tables

**Table 1:** The characteristics of groups under investigation.

Group	*n*	Males	Females	Median age (range)
Donors	28	13	15	62 (24–72)
Gastric cancer patients	41	20	21	68 (28–83)
